# Examining Latin America’s Transition to a Circular Economy for Plastics

**DOI:** 10.1007/s00267-025-02298-9

**Published:** 2026-01-10

**Authors:** Lina Raquel Rodríguez-Meza, Felipe Romero-Perdomo, Miguel Ángel González-Curbelo

**Affiliations:** 1 Departamento de Ciencias Básicas, Facultad de Ingeniería, Universidad EAN, Bogotá D.C., Colombia; 2 Corporación Colombiana de Investigación Agropecuaria-AGROSAVIA, Mosquera, Cundinamarca Colombia

**Keywords:** Circularity, Plastics treaty, Plastic pollution, Plastic waste management, Plastic governance, Policy analysis

## Abstract

In light of global efforts to advance a circular economy for plastics, this study examines Latin America’s transition through three core objectives. First, it analyzes secondary data on plastic production and consumption and the generation, mismanagement, and transboundary trade of plastic waste. Second, it scrutinizes government-led initiatives across the region based on official policy documents. Third, it conducts a SWOT analysis, evaluating the initiatives’ strengths, weaknesses, opportunities, and threats to assess the current landscape of circular product design and business models, as well as their potential to mitigate the environmental impacts of the triple planetary crisis. Findings reveal that plastic production, consumption, and waste are steadily increasing in the region, while waste management and sustainable trade remain insufficient. The circular economy for plastics has gained traction through national strategies, roadmaps, and legal instruments. Its adoption has been notable in Chile and Uruguay, but negligent in several countries. Governments are supporting research into recycled materials and polymer innovation, yet policy gaps persist around microplastics and harmful additives in plastic product design. Most initiatives prioritize circular supply chains and resource recovery business models, while giving limited attention to other models and the underlying drivers and barriers. Furthermore, initiatives often address plastic pollution with weak linkages to climate change and biodiversity loss. This research strengthens the understanding and implementation of actions positioning circular design as pivotal to reducing plastic waste at the source, circular business models as catalysts for low-carbon economies, and the fight against the triple planetary crisis as an environmental objective of circular economy initiatives.

## Introduction

The growing problem of plastic management is a pressing global issue. Virgin plastic production, plastic made from newly extracted fossil resources rather than recycled materials, has increased from 1.5 million tons per year in 1950 to 413 million tons in 2023 and is projected to exceed one billion tons per year by 2050 (Plastics Europe, 2024). Unlike recycled plastic, virgin plastic requires more energy and raw materials, contributing directly to greenhouse gas emissions and resource depletion. Despite growing recycling efforts, plastic production continues to outpace global waste management capacity, contributing to an estimated 20 million tons of plastic leakage into the environment each year (Pilapitiya and Ratnayake, 2024). The economic costs of plastic pollution are largely associated with losses in marine tourism, reductions in income from fishing and aquaculture, expenses for waste management technologies and systems, and decreases in human benefits derived from ecosystem services. These impacts amount to annual losses greater than $2.5 trillion (Nikiema and Asiedu, 2022). The profound systemic challenge posed by plastics accelerates the triple planetary crisis: climate change, pollution, and loss of biodiversity, recognized as interconnected threats to human health and sustainable development (Almroth et al., 2022).

The circular economy represents one of the responses with the potential to face this growing problem, transforming the linear economic model based on the plastics industry’s take-make-dispose to improve its sustainability (Geissdoerfer et al., 2020). The circular economy seeks to reduce waste and maximize the use of resources by designing products that can be reused, repaired, and recycled (Shi et al., 2024). The relevance of the circular economy lies in its potential to decouple the use of plastic from the consumption of finite resources, create reuse models, and redesign and replace environmentally problematic plastic packaging with reusable, recyclable, or compostable alternatives, among other impacts (Ayassamy, 2025; Cader et al., 2024). By 2040, adopting a circular economy could potentially reduce the annual amount of plastics entering the oceans by 80%, reduce greenhouse gas emissions by 25%, save $200 billion a year, and create 700,000 net new jobs (Ellen MacArthur Foundation, 2023).

Governments are responding politically to the circular economy in plastics management, adopting various actions worldwide. In China, the circular economy law promotes the reduction, reuse, and recycling of materials (Sun et al., 2022). The Netherlands and Denmark are moving forward with national strategies that include taxes on non-recyclable plastics and ambitious recycling targets. Germany, a pioneer in waste management, implements the principle of Extended Producer Responsibility (EPR) to ensure that manufacturers take responsibility for managing plastic waste (Perissi, 2025). In the United States, several cities and states are banning single-use plastics and promoting recycling programs. The United Kingdom, for its part, has introduced a tax on plastic packaging that contains less than 30% recycled material, thus encouraging the use of recycled plastics (Pilapitiya and Ratnayake, 2024). These examples are relevant to Latin America because they offer successful approaches that can inform the design and implementation of similar policies adapted to regional conditions.

Latin American countries also face challenges in effectively managing plastics and are integrating the circular economy into their new policies (Schröder et al., 2020). The circular economy is particularly well-suited to the region because it promotes resource efficiency, reduces dependency on imported raw materials, and supports job creation in recycling and reuse sectors, which are critical factors for many Latin American economies. Chile has implemented the EPR law, which forces producers to manage the life cycle of their products, including plastics (Government of Chile, 2021). Colombia launched the National Circular Economy Strategy, which aims to reduce the use of single-use plastics and promote the recycling and reuse of plastic materials (Government of Colombia, 2019). Ecuador is promoting the circular economy through policies that encourage innovation in recycling and plastic waste management, with specific programs that seek to involve both the private sector and civil society in the transition to a more sustainable circular economy (Government of Ecuador, 2021).

Although Latin America has not remained indifferent to the plastic crisis in the context of the circular economy, research efforts have largely focused on developed countries (O’Halloran, 2024; Hendrickson et al., 2024; Eckert et al., 2024; Amadei et al., 2023). Ospina-Mateus et al. (2023) pointed out the underrepresentation of the region in the literature. Existing studies tend to focus on multiple industries or those unrelated to plastics, specific countries or processes, and often neglect the policy aspects (Salvador et al., 2022; Aguilar et al., 2022; Betancourt Morales and Zartha Sossa, 2020). Among the few available studies, Rodríguez-Meza et al. (2025) reported that several governments in the region are promoting a plastic recycling-based economy without investing in advanced recycling technologies. They identified gaps that need to be addressed, such as regulating microplastics and hazardous additives within circular design frameworks, promoting circular business models, and adopting a robust approach to environmental impact, among others. There are numerous ongoing debates about how to achieve a circular economy in Latin America, at what level, and the role of governments in this process (Henao-Hincapié et al., 2024).

To provide a regional overview of Latin America in this context, this study adopted a multi-objective approach. The first objective was to examine the status of plastic production and consumption, as well as the generation, mismanagement, and trade of plastic waste using secondary data. The second objective was to map the government-led initiatives aimed at advancing a circular economy within the plastic industry. Building on these inputs, the third objective was to qualitatively assess the strengths, weaknesses, opportunities, and threats (SWOT), serving as a situational diagnosis of the region across three dimensions: circular design, circular business models, and the triple planetary crisis. Table S1 describes these three dimensions of analysis.

## Methodology

### Plastic Management Data

Secondary data associated with plastic management in Latin America were collected using official government websites, including those of the presidency, congress, and environment ministries. The analysis included the following countries in the region: Argentina, Bolivia, Brazil, Chile, Colombia, Costa Rica, Cuba, the Dominican Republic, Ecuador, El Salvador, Guatemala, Haiti, Honduras, Mexico, Nicaragua, Panama, Paraguay, Peru, Uruguay, and Venezuela. Moreover, reports from key organizations involved in plastics management and the circular economy were reviewed, such as the Organization for Economic Cooperation and Development (OECD), the Ellen MacArthur Foundation, the Platform for Accelerating the Circular Economy, and the United Nations Environment Program (UNEP), among others.

Search terms and keywords were adapted to the official languages of each country (Spanish, English, and Portuguese) and centered on two main themes:

I. Plastics, including terms such as “chemical sector”, “chemical industry”. “plastics sector”, “plastics industry”, and “plastics economy”.
II. Economy, with terms like “linear economy”, “fossil fuel-based economy”, “circular economy”, “circular bioeconomy”, “bio-based economy”, and “bioeconomy”.

These terms were used individually and in various combinations to ensure comprehensive coverage and facilitate the identification of circular economy plans in each country (Dace et al., 2024). The characterization of plastic management in Latin America focused on five key areas: (i) plastic production, (ii) plastic consumption, (iii) plastic waste generation, (iv) estimation of inadequately managed plastic waste, and (v) plastic recovery trade. These areas were selected because they represent critical stages in the plastic value chain, from material input to end-of-life outcomes, allowing for a comprehensive assessment of production pressures, consumption patterns, environmental leakage, and recovery efforts.

### Identification of Government Initiatives

Government documents on circular economy initiatives across Latin America were collected from official state institution websites. These initiatives encompassed plans, roadmaps, policies, laws, regulations, programs, strategies, and frameworks (Table S2). If a country lacked a dedicated circular economy initiative, official documents referencing the plastics industry, even if not exclusively focused on circularity, were included. Documents had to be official, national, and explicitly mention both the circular economy model and its application to plastics.

The search covered the same 20 countries using the previously described keyword strategy. Document content was reviewed and systematized according to the guidelines in the following subsection. Graphs were performed to display the number of circular economy initiatives by type and by year.

### SWOT Analysis

A qualitative SWOT analysis was used. This methodology is a strategic planning technique implemented in terms of strengths, opportunities, weaknesses, and threats, originally used by companies to improve their competitiveness (Stacey, 1993), but more recently used to analyze sustainable development contexts (Qaiser, 2022; Kaymaz et al., 2022). Unlike other tools such as political, economic, social, technological, environmental, and legal analysis (PESTEL) or value chain analysis, SWOT was selected because it allows for a structured yet adaptable assessment of complex policy environments, enabling the integration of internal and external factors that influence circular economy initiatives. Furthermore, PESTEL analysis primarily examines the overarching external influences, and this study does not follow the plastic value chain framework but addresses three understudied dimensions. The SWOT analysis assessed whether key aspects of governments’ circular economy policy responses can facilitate (strengths and opportunities) or hinder (weaknesses and threats) a positive effect on plastics management. While strengths and weaknesses are linked to the internal advantages and disadvantages of various actions, opportunities and threats are linked to the possible external positives and negatives of their implementation (Stacey, 1993).

The assessment focused on circular design, circular business models, and environmental impacts of the triple planetary crisis, which were referred to as dimensions in this study. Circular design was examined by evaluating whether government initiatives support recycled plastics and new (bio)polymers to reduce reliance on fossil raw materials, restrict harmful chemicals, regulate microplastics, establish circular indicators, and introduce economic incentives. Circular business models were analyzed based on whether initiatives promote five key models: circular supplies, product-as-a-service, product life extension, sharing platforms, and resource recovery. Finally, the study assessed whether these initiatives address environmental impacts related to the triple planetary crisis: pollution, biodiversity loss, and climate change. These aspects were selected due to their significance in critical value chain processes, their demand for stakeholder engagement, and their impact on future environmental sustainability. Moreover, they have been recognized as gaps even in leading countries advancing plastic circularity (Rodríguez-Meza et al. 2025; Arora et al., 2024; Dijkstra et al., 2020).

After reviewing the initiatives, specific sentences referring to the three dimensions were extracted. The contribution of the sentences was then analyzed and discussed among the authors by country, by dimension, and by SWOT component. Mention of a dimension in the extracted text served as an indicator that the country had proposed actions in that regard.

## Results

### Panorama of Plastics Management in Latin America

#### Plastics Production

The data shows that plastic production began in the early 20th century with the development of industrial polymers derived from fossil materials. It expanded rapidly, growing from 1.5 million tons in the 1950s to 200 million tons by 2002, driven by the increased use of ethylene, propylene, and styrene (Plastics Europe, 2024). This period marked a 133-fold surge in production, accompanied by rising environmental concerns. Global output surpassed 300 million tons in 2014, reaching 368 million tons in 2019, an 84% increase in less than two decades (Syberg et al., 2021). Although the COVID-19 pandemic briefly slowed production in 2020, the growing demand for single-use plastics led to a rebound, pushing output to 394 million tons in 2021, 400 million tons in 2022, and 413 million tons in 2023 (Plastics Europe, 2024).

Asia, particularly China, leads global plastic manufacturing, accounting for 32% of total production. China alone produces between 6 and 12 million tons of plastic goods each month. Other Asian regions contribute 19%, followed by North America at 17%, Europe at 14%, and the Middle East at 9%. Latin America, though data is less comprehensive, produced over 15 million tons in 2022, representing 4% of global output (Plastics Europe, 2024).

#### Plastics Consumption

The dynamics of plastic consumption exhibit that it has grown dramatically, increasing 180-fold between 1950 and 2018, closely following production trends. The largest consumers are North America (21%), China (20%), and Western Europe (18%) (Ryberg et al., 2019). Global plastic consumption is expected to reach 1231 million tons by 2060, with the fastest growth occurring in developing nations across Africa and Asia (Ambituuni et al., 2025; Pilapitiya and Ratnayake, 2024).

Latin America represents 8% of global plastic consumption (OECD, 2022). Per capita consumption rose from 7 kg in 1980 to 21 kg in 2005 and is now estimated at 37 kg per person. In 2021, per capita consumption by country was: Mexico (55 kg), Chile (54 kg), Argentina (42 kg), Brazil (37 kg), Peru (33 kg), and Colombia (28 kg). These figures are close to the global average of 45 kg per person per year, reflecting a steady upward trend in plastic consumption across the region (Bianco et al., 2021).

#### Generation of Plastic Waste

Global plastic waste generation has doubled between 2000 and 2019, reaching 353 million tons. Of this total, 63% came from single-use plastics, with packaging accounting for 40%, consumer goods for 12%, and clothing and textiles for 11% (OECD, 2022). Projections indicate that plastic waste could triple to 1014 million tons by 2060, with environmental leakage rising to 44 million tons per year and plastic accumulation in water bodies tripling. In 2019, per capita plastic waste generation was 220.5 kg in the United States, 117.9 kg in Canada, and 121.6 kg in OECD European Union countries (Pilapitiya and Ratnayake, 2024).

Latin America reflects a similar trend. The region generated 232.8 million tons of solid waste in 2020, with plastic waste accounting for 28.8 million tons, or 12.4% of the total. The largest plastic waste producers that year were Brazil, Mexico, Argentina, and Colombia (Brooks et al., 2020). Brazil exhibited plastic waste generation nearly double that of Mexico, which, in turn, was double that of Argentina (Table S3). Plastic waste generation in Colombia accounted for 63% of that in Argentina. While there were slight decreases in plastic waste generation from Venezuela to other countries, plastic waste generation per capita reveals Argentina as the highest, followed by Brazil and Mexico (Table S3). Per capita plastic waste generation ranged between 41 and 35 kg per person in Panama, Uruguay, Venezuela, Chile, the Dominican Republic, Paraguay, and Ecuador, while the remaining countries fell below 35 kg per person (Brooks et al., 2020).

#### Estimation of Inadequately Managed Plastic Waste

The generation of plastic waste underscores the importance of proper management. Between 1950 and 2018, an estimated 6300 million tons of plastic waste entered ecosystems and open-air landfills globally, with ~80% of this waste being inadequately managed (Kumar et al., 2021). Projections indicate that by 2050, the volume of mismanaged plastic waste could reach 12,000 million tons (OECD, 2022).

In Latin America, 8.4 million tons of plastic waste were inadequately managed in 2020, with an expected increase to 10.3 million tons by 2050. Coastal countries in the region contributed 3.7 million tons of plastic waste in 2020, with the potential to enter the oceans (Brooks et al., 2020). The Latin American countries with the highest amounts of improperly managed plastic waste in 2020 were Brazil, Mexico, Argentina, and Peru (Table S4). However, per capita calculations reveal a different trend, with three Central American countries, the Dominican Republic, Nicaragua, and Guatemala, leading the rankings, followed by Chile and Panama (Table S4; Brooks et al., 2020).

#### Plastic Waste Trade

The global trade of plastic waste expanded significantly between 1995 and 2010, with imports and exports rising from ~3 million tons per year to nearly 16 million tons, a level that remained steady until 2017 (United Nations Conference on Trade and Development [UNCTAD], 2021). However, trade declined notably as China in 2018, a major importer, banned certain plastic waste imports due to filling its Jiangcungou mega-dump ahead of schedule. This led countries like the United States to seek new importers in Southeast Asia, Turkey, Africa, and Latin America, particularly Mexico (Brown et al., 2023).

The COVID-19 pandemic further impacted global trade, reducing the plastic waste market to around 6 million tons per year in 2020 and 2021. During this period, the top exporters of plastic scrap were Germany, Japan, and the United States, while the main importers were the Netherlands, Turkey, and Malaysia (Observatory of Economic Complexity, 2021).

**Fig. 1:**
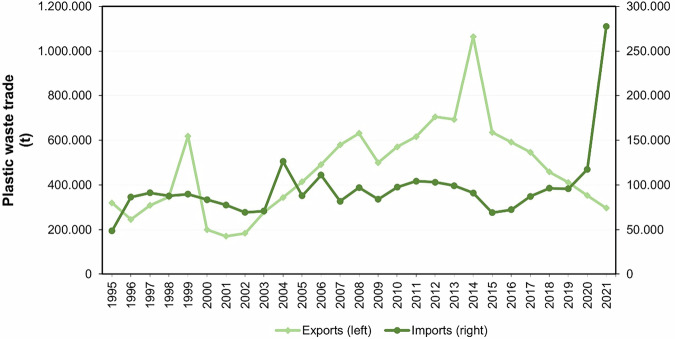
Plastic waste trade in Latin America. Own elaboration based on data from UNCTAD (2021). The right *Y*-axis of the graph corresponds to imports, and the left *Y*-axis corresponds to exports. The raw data for this figure can be seen in Table S5

Latin America’s contribution to global plastic waste exports was higher between 1995 and 1999, averaging 11.7%, compared to the period from 2000 to 2021, where it averaged 4.6%. The peak participation occurred in 1999 at 14.7%, while the lowest was recorded in 2022 at 3.3%. From 2000 to 2014, a notable increasing trend was observed, with exports rising from 200,618 tons to 1,064,648 tons of plastic waste, although minor declines were noted in 2001, 2009, and 2013 (Fig. 1). However, since 2015, exports have consistently decreased year after year (UNCTAD, 2021).

Latin America’s share of global plastic waste imports varied over the years, accounting for 1.5% (48,836 tons) in 1995, 1.4% (83,638 tons) in 2000, 0.58% (97,591 tons) in 2010, and 1.8% (117,441 tons) in 2020. The region saw a peak in imports in 2004, reaching 126,242 tons. By 2021, plastic waste imports had risen to 277,555 tons, representing 4.5% of global imports, the highest share recorded to date (UNCTAD, 2021).

Mexico stands as the leading exporter and importer of plastic waste in the region (Table S6). Mexico exported 11.2 million tons and imported 1.3 million tons from 1988 to 2018 (Brooks et al., 2020). Following Mexico in exports are Argentina, Ecuador, the Dominican Republic, El Salvador, Brazil, and Chile. Regarding imports, Brazil, Colombia, and Chile rank after Mexico. Peru recorded the lowest amount of plastic waste exports, while Argentina had the lowest imports (Brooks et al., 2020).

Latin America has witnessed a critical influx of plastic waste from the United States, with exports soaring by over 100% in the first half of 2020 alone. During this period, the United States shipped 44,173 tons of plastic waste to fifteen Latin American nations (Wang et al., 2020).

The plastic waste trade in Latin America reveals high exports and low imports, indicating insufficient recycling capacity within the region. Latin America has the lowest recycling rate globally, standing at just 4.5%, compared to the global average of 13.5%. While the circular economy aims to eliminate waste rather than focus solely on recycling, a lack of recycling infrastructure can hinder the transition to a circular system (Kaza et al., 2018).

### Current State of Political Responses for the Circular Economy of Plastics in Latin America

**Fig. 2:**
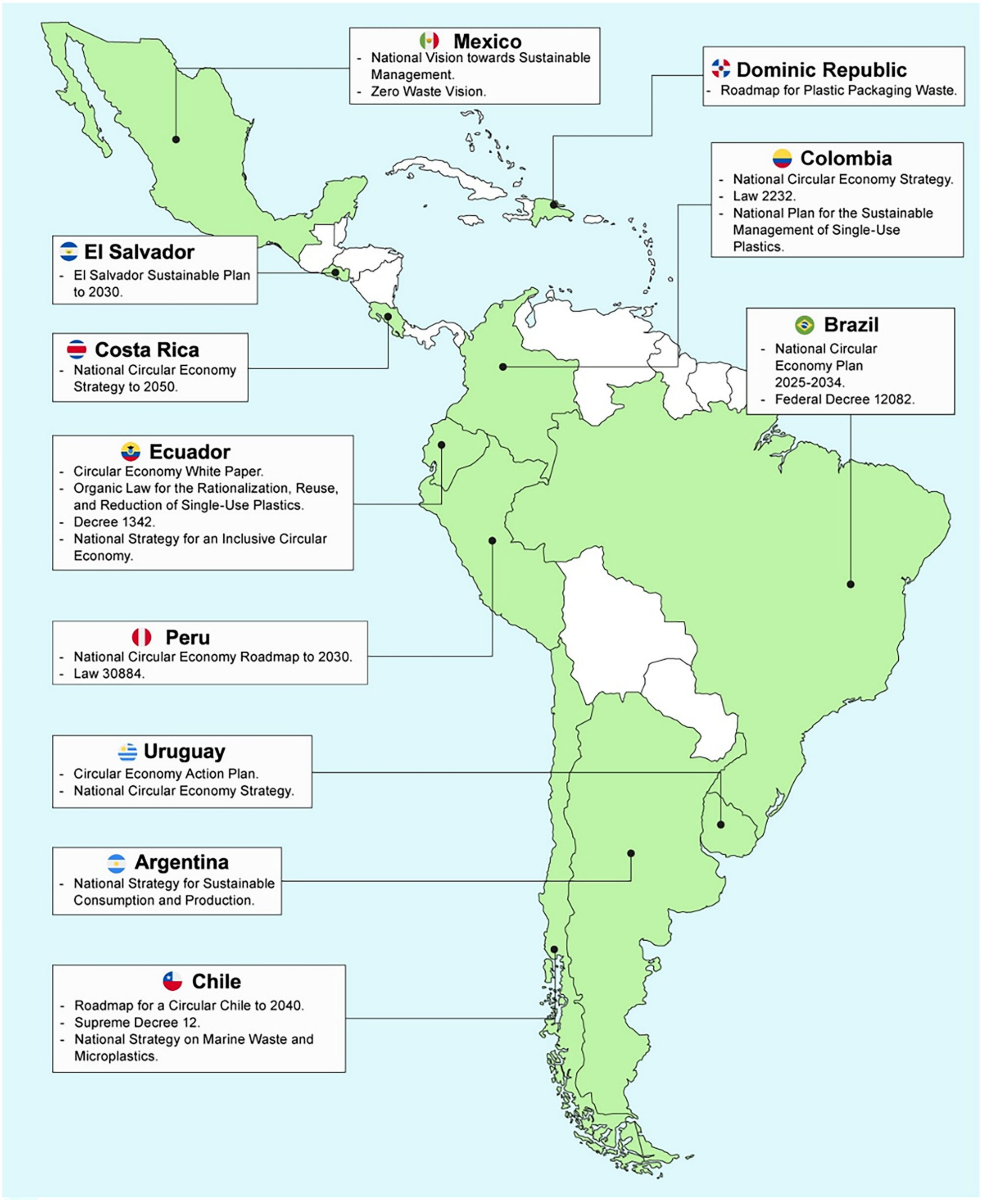
Circular economy initiatives for plastics in Latin America

The results show that the region’s initiatives take the form of plans, roadmaps, laws, strategies, and decrees. The most common are strategies, roadmaps, and plans (Fig. S1). The introduction of these initiatives began in 2018, peaking in 2021, followed by another surge in 2024 (Fig. S2). To date, 11 countries have launched government-led efforts to promote a circular economy for plastics: Argentina, Brazil, Chile, Colombia, Costa Rica, Ecuador, El Salvador, Mexico, Peru, the Dominican Republic, and Uruguay (Fig. 2). Among them, Chile, Uruguay, Costa Rica, and Ecuador stand out for their dedicated circular economy initiatives, which emphasize clear objectives, broad scope, and robust monitoring and evaluation measures. These countries are actively aligning their policies with global trends observed in developed nations. The specific initiatives undertaken by 11 countries are detailed in Table S7.

In Latin America, 9 countries have not officially launched government initiatives. These are Bolivia, Cuba, Guatemala, Haiti, Honduras, Nicaragua, Panama, Paraguay, and Venezuela. Table S8 summarizes the efforts closer to the circular economy that these countries have reported. Additionally, there are regional initiatives led by various organizations. Three of the most prominent ones are the Circular Economy Coalition for Latin America and the Caribbean, the Circular Plastics Program of the Americas, and the Plastics Pact Network.

Of the three dimensions assessed, countries with circular economy initiatives are focusing primarily on circular design (Table 1). While there is broad support for the use of recycled plastics and the development of new (bio)polymers, there are gaps in the management of microplastics and harmful chemicals. Chile, Uruguay, Ecuador, and Colombia stand out for their circular design actions. Second, countries are promoting circular business models for plastics, with circular sourcing and resource recovery being the most common. Uruguay, Chile, and Costa Rica actively promote these models, while other countries reference them more indirectly, often linking them to industrial symbiosis. Third, the Triple Planetary Crisis is included only weakly and inconsistently. Chile and Costa Rica have the strongest measures to address it. Across initiatives, plastic pollution receives the most attention, followed by climate change and biodiversity loss. Additionally, many initiatives lack a unified vision and often repeat the same discourse without establishing measurable objectives or indicators.

**Table 1:**
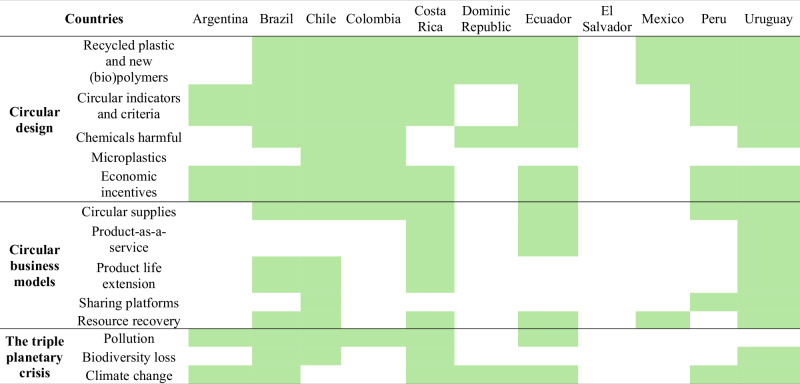
Content analysis of circular economy initiatives in terms of circular design, circular business models, and the triple planetary crisis

The green color indicates that at least one initiative from the country proposes actions. This table was developed based on Table S9

When comparing countries across all dimensions, Chile and Uruguay stand out. They share several common actions, including a clear vision with measurable objectives and progressive goals to ensure that all plastic products in their markets are fully recyclable or degradable. They connect their circular economy initiatives with other existing initiatives, such as national plans on climate change, the bioeconomy, and renewable energy, among others. Furthermore, they define cross-sector action plans with a strong emphasis on plastics. They also adopt a complete value chain approach to plastics, quantifying material flows. Additionally, they place importance on innovation, culture, regulation, and governance.

### SWOT Analysis Developed

#### Circular Design

The main strength of the region’s circular economy initiatives in circular design is the promotion of the reuse of recycled plastic materials and the creation of bio(polymers) (Table 2). To support this, countries are investing in research on innovative materials and encouraging eco-design practices. Some are also working, though to a lesser extent, on establishing durability, reuse, and repair metrics. Several nations are exploring ways to incentivize eco-design adoption among businesses, implement training programs, and create regulatory and reference frameworks. Meanwhile, other countries have opted to ban certain single-use plastics, discouraging the use of petrochemical polymers from the design stage.

**Table 2:** SWOT analysis of circular design, circular business models, and the triple planetary crisis in the circular economy initiatives of Latin American governments

Aspect	SWOT analysis
Circular design	*Strengths:* Promotion of the reuse of recycled plastic materials; support for research into new bio(polymers); banning certain single-use plastics.
	*Weaknesses:* Disagreements on circular design criteria; limited scope for 10R* strategies in eco-labels; no emphasis on microplastics and harmful additives; weak connection between design and recycling technological capabilities; few economic incentive initiatives; production of fossil polymers without environmental analysis.
	*Opportunities:* Emerging markets around recycled products and bioplastics; advances by pioneering countries; academic programs; taxes on petrochemical plastics; restrictions or reduction targets on problematic polymers; standardizing product designs internationally; growing research into harmful chemical microplastics.
	*Threats:* Petrochemical industry influence on governments; the passivity of countries; high innovation costs; greenwashing.
Circular business models	*Strength:* High support for business models such as circular supply and resource recovery.
	*Weaknesses:* Scarce support for circular business models such as product life extension, product-as-a-service, and exchange platforms; lack of harmonization of business models; lack of tax incentives for companies; medium or low approach to providing public financing.
	*Opportunities:* Accelerate the circular transition; global trend; access to new markets.
	*Threats:* High innovation costs; high risk aversion in the business sector; market penetration remains limited.
The triple planetary crisis	*Strength:* Giving prominence to plastic pollution.
	*Weakness:* Negligent attention to biodiversity loss and climate change; inconsistencies and lack of harmonization in regulations and policies; few measurements of poorly managed plastics and their harmful effects; scarce actions to mitigate the release of microplastics; soft measures that rely on voluntary compliance.
	*Opportunities:* Environmental education; Global Plastics Treaty; increasing evidence on negative impacts; other international environmental agreements; growing citizen demand for sustainable policies; prioritizing human health.
	*Threats:* Political and social instability; corruption; continued excessive production and consumption of plastics; low political credibility in environmental scientific evidence.

^*^10R refuse, rethink, reduce, repair, restore, remanufacture, refurbish, repurpose, recycle, and recover

Latin America faces several weaknesses in implementing circular design principles. The current eco-labeling systems primarily focus on recycling, with limited emphasis on the broader 10R strategies. There is also a weak connection between product design and the technological capabilities needed for efficient recycling, which is crucial for determining quality and recycling rates, as well as guaranteeing the circulation of recycled plastic as an input. Governments largely overlook the risks posed by microplastics and harmful additives, as they have yet to introduce regulations addressing these issues in product design. Moreover, environmental impact assessments fail to account for the primary production of fossil-based polymers, leaving a critical gap in covering the entire life cycle of plastics.

Many governments also fail to establish economic incentive initiatives for new circular materials and technologies to be competitive in the market against their fossil counterparts. While regulatory instruments are the most advanced lever, in financial terms, they are still largely nascent in the region. These are the instruments that reduce capital costs and catalyze large-scale private investment. Brazil, Chile, Colombia, Ecuador, and Uruguay place the greatest emphasis on financial incentives. They all agree on seeking international impact investment funds, as well as evaluating the cost-benefit ratio of tariff barriers and incentives. They plan to implement predictable public subsidies, tax breaks, and scalable blended financing; however, concrete examples of these measures have yet to emerge.

Among the opportunities for countries are markets that can be created around recycled products and bioplastics, exceeding 7 billion dollars (Mousavi et al., 2024). The advances made by pioneering countries in new materials, metrics, and selection guidelines for design can serve as a point of reference (Rosenboom et al., 2022). Furthermore, countries can agree internationally on product design standards (Arora et al., 2024). Investing in circular economy-based educational programs can influence consumer behavior, strengthen the academic sector, and drive innovation in product design. Scientific research on microplastics is growing rapidly, revealing their widespread environmental and health impacts (Gündoğdu et al., 2024). Studies have also identified numerous harmful chemicals in plastics that are released throughout their life cycle (Wagner et al., 2024). In addition, policymakers have powerful tools at their disposal, such as taxing plastics derived from petrochemicals and, above all, imposing limits or reduction targets on problematic polymers (Tilsted et al., 2023).

The influence of the petrochemical industry on government decision-makers can greatly affect the circular transition of plastic design. Countries with economies based on fossil resources that convert large quantities of oil and gas into plastic products promote measures that hinder circular development (Arora et al., 2024). Similarly, the lack of commitment by countries to act and launch circular economy initiatives is a limitation. High innovation costs are one of the best-known bottlenecks. Some circular technologies are available on the market, while others are still in development (Solis and Silveira, 2020). However, as the economic benefits are not yet abundant, the prices of these technologies remain high. Greenwashing is an image-laundering technique used by many companies to make their products appear environmentally friendly when they are not (Choudhury et al., 2024). Undoubtedly, the behavior of companies and the rigor of state actions are critical to giving legitimacy to the transition process. Finally, there is still a lack of political credibility regarding environmental scientific evidence, which minimizes the reality of the plastic crisis and threatens the well-being of the planet for future generations.

#### Circular Business Models

The SWOT analysis exhibits that many countries are showing strong support for circular business models (Table 2). A strength shared by few countries is developing a national sectoral assessment that allows for mapping existing circular business models for plastics. This identifies practices, technologies, barriers, and opportunities, and also compares them with the situation of the most prominent countries in this context, such as those in the European Union. Strategies are then defined to develop circular markets with potential, depending on the country’s development conditions. However, a notable weakness is the limited support for business models focused on product life extension, product-as-a-service, and sharing platforms. Additionally, there is a lack of alignment in business models, as some countries overlook the five widely recognized typologies included in this paper. Government support through public funding and tax incentives for businesses also remains moderate to low.

Opportunities to promote circular business models include involving more stakeholders in the value chain, identifying financing and investment opportunities, fostering international collaboration, and beginning to apply circular criteria in government processes and public procurement (Geissdoerfer et al., 2020). Since circular business models are gaining traction globally, countries that adopt them will have greater access to international markets. Chile provides a clear example of how these opportunities can translate into action. The country has focused primarily on plastic resource recovery and circular supply business models. Its EPR law, single-use plastics law, and strong public-private collaboration have been key to opening circular markets. Moreover, the government, advised by the Netherlands, has promoted circular public procurement and consumer behavior, while working to develop suitable financing structures. Although still in early stages, Chile is already seeing both economic and environmental value from these efforts (Rodríguez-Meza et al., 2025).

The threats that can most affect circular business models are the high costs of innovation and the business sector’s cautious approach to risk. These challenges highlight the importance of introducing economic incentives to support industries and maintaining close collaboration between governments and businesses to drive changes in business models, strategies, technologies, and workforce training. To overcome risk aversion, governments could offer tax breaks, innovation grants, or preferential loans for circular initiatives, while also creating pilot programs that demonstrate the economic viability of circular approaches (Urbinati et al., 2020).

#### The Triple Planetary Crisis

The results show that governments are placing increasing importance on plastic pollution over time, relying more on global data than local data (Table 2). For example, Chile promotes collaboration between academia, the public and private sectors, and civil society to guide decision-making, assess impacts, identify solutions, and share knowledge about the environmental effects of plastics. Uruguay aims to incorporate cross-cutting considerations of climate change and biodiversity into the formulation of circular economy projects to optimize access to financing.

As a weakness, most governments are not fully addressing the triple planetary crisis, often neglecting biodiversity loss and climate change. This oversight undermines the development of systemic responses, limits the region’s capacity to meet climate and biodiversity targets, and increases the risk of irreversible environmental degradation (Almroth et al., 2022). It is evident in two main ways: in most cases, these issues are simply not mentioned, while in others, initiatives focus on plastic pollution but show only vague interest in other environmental impacts. Furthermore, these efforts fail to engage with key international bodies such as the Intergovernmental Panel on Climate Change and the Intergovernmental Science-Policy Platform on Biodiversity and Ecosystem Services. Other gaps in the region include limited attention to measuring the effects of mismanaged plastics, reducing microplastic emissions, and aligning technical and environmental regulations and policies. Additionally, many measures depend on voluntary compliance rather than enforceable regulations.

There are clear opportunities to strengthen collaboration around the Global Plastics Treaty and deepen commitment to other international environmental agreements like the Basel, Stockholm, Rotterdam, and Kunming-Montreal Conventions, the Paris Agreement, and the Rio Declaration (Vince et al., 2024). These global frameworks have proven effective in other regions; for example, in the European Union, the Circular Economy Action Plan and alignment with international climate and chemical agreements have catalyzed national legislation, investment in sustainable technologies, and waste reduction efforts (Eckert et al., 2024). Rising public demand for sustainable policies, along with increasing evidence of the harmful effects of plastics on both the environment and human health, is pushing plastic-related issues higher on political agendas.

Major threats persist, including political and social instability and corruption. Political stability in Latin America remains fragile compared to Europe. For instance, in Brazil, frequent changes in environmental leadership and shifting political priorities have delayed or reversed progress on plastic and biodiversity policy implementation (Bersch and Lotta, 2024). The credibility of scientific evidence on environmental issues remains weak and is gradually diminishing, hindering strong decision-making. The most pressing threat is the continued high production and consumption of plastics, which risks overwhelming the planet with plastic waste (Arora et al., 2024).

## Discussion

### Plastic Management and Government Responses

This research reports that Latin America continues to encounter difficulties in plastic management. Plastic production, consumption, and waste generation continue to increase, while management remains inadequate, and there is limited sustainable progress in plastic waste trade. Likewise, there are significant gaps in understanding plastic stocks and flows. Addressing these gaps is essential for designing effective policy interventions: knowing how much plastic is produced, where it accumulates, how it moves through the economy, and how it leaks into the environment enables governments to target interventions, optimize waste management systems, prioritize high-leakage sectors, and track the effectiveness of circular economy strategies. Given that the situation in Latin America mirrors a global trend, tackling the plastics crisis undeniably requires coordinated efforts both internally and externally by and between nations (Hossain et al., 2022). This need for collaboration is evident in the negotiation of the legally binding Global Plastics Treaty (Vince et al., 2024).

The study finds that the region is taking steps through government efforts toward a circular economy to address the negative impacts of improper plastics management. However, it faces challenges in the disparity in countries’ responses, with almost half of the region, mainly in Central America, maintaining a passive attitude due to the lack of circular economy initiatives. Some countries delve deeper into the plastic crisis and propose industry-specific actions, while others establish cross-sectoral efforts with limited relevance to plastics. Although most countries are focusing on improving plastic waste management capacity, projections suggest that this alone will not be enough to manage the increasing rate of plastic waste (Borrelle et al., 2020). Additionally, several countries have not defined measurable goals, monitoring, and follow-up actions, as well as being non-specific about the economic instruments that governments plan to use (e.g., taxes and fees, subsidies, deposit return systems, and early disposal fees).

Barrie et al. (2024) highlighted that circular economy initiatives require a robust monitoring and evaluation system supported by an institutional governance system that extends beyond short-term political cycles. Among their other findings, they observed that initiatives are often managed by one or two government ministries, leading to a lack of a comprehensive, whole-of-government approach. Establishing inter-ministerial commissions could help overcome this by facilitating policy alignment, streamlining regulatory frameworks, pooling technical and financial resources, and ensuring consistency across ministries in implementing circular strategies. This would foster a more coherent, whole-of-government approach to the circular economy, improving effectiveness and long-term impact.

### Towards a Circular Design for Plastics

The findings indicate that Latin American initiatives might be pushing a semi-circular model for plastic product design instead of fully embracing circular economy principles. Semi-circular models typically focus on isolated actions like recycling or substituting materials without addressing the entire lifecycle of products. Merely promoting new (bio)plastics and the reuse of recycled plastic materials is insufficient if both their circular properties and economic incentives are partially addressed and if the risks associated with both microplastics and harmful additives, and the dependence on fossil fuels are overlooked. This could lead to a market where circular plastics are not competitive compared to traditional plastics, becoming just another option while retaining some negative aspects, and ultimately generating an unmanageable waste mix. Countries should ensure that all plastic products on the market are either fully recyclable or degradable, closing resource loops as much as possible by applying the 10R framework across the value chain. Canada and Georgia suggest standardizing circular parameters of plastic product designs internationally (Arora et al., 2024).

Supporting bioplastics requires rigorous decisions. Governments should not assume that bioplastics are always more environmentally friendly than fossil-based plastics. While they often have a lower carbon footprint and better circular properties, they can also cause environmental issues like eutrophication, acidification, and competition with food production (Nikiema and Asiedu, 2022). Policy decisions should therefore be guided by scientific evidence and ongoing dialogue with researchers. The European Union’s approach, for instance, focuses on ensuring that bioplastics offer real environmental benefits throughout their lifecycle, are clearly labeled, meet rigorous standards, and are properly managed at the end of their life to prevent pollution and promote a circular economy. The 2022 Policy Framework on Bio-Based, Biodegradable and Compostable Plastics, the new Regulation 2025/40 on Packaging and Packaging Waste, and the Consumer Empowerment Directive 2024/825 are particularly significant recent legislative developments boosting bioplastics (Paloniitty and Ala-Lahti, 2024).

Making choices about recycled plastics also demands careful consideration. Policymakers need to choose an appropriate scope and consider many characteristics when designing rules for reuse. These include target material, target group, target product, system boundaries, and recycling technological capabilities (Maeder and Fröhling, 2024). Similarly, governments must address factors like the price difference between recycled and virgin materials, the cost disparity between recycling and alternative methods, and the high volume of plastic imports (Solis and Silveira, 2020).

The regulation of microplastics and harmful additives in the design of plastic products is a deficiency in Latin America’s circular economy initiatives. Failing to address the risks associated with microplastics can lead to widespread environmental contamination, bioaccumulation in food chains, and long-term human health impacts (Winiarska et al., 2024). The European Union has prioritized its regulation, including them as contaminants within the regulatory framework. Regulation could cover prohibiting both the intentional addition of microplastics to consumer products and the sale of products that release microplastics to a large extent (Gündoğdu et al., 2024). For chemical additives, transparency, harmonization, and simplicity in chemical compositions are required; those that are highly dangerous to the environment and human health must be prohibited, and all additives must be subject to regulation (Wagner et al., 2024). In response, the creation of a technical and testing body responsible for carrying out regular testing of polymers, chemical additives, and the presence of microplastics has been suggested in the Global Plastics Treaty (Arora et al., 2024).

### Promoting Circular Business Models

This paper reveals that Latin America has paid some attention to circular business models for plastics, but several aspects require further study. The region has focused on circular supply and resource recovery models to generate mutual benefits for businesses, society, and the environment. However, there’s still a lack of understanding about the different types of circular models, the factors that could encourage greater adoption, and how to overcome existing barriers for businesses. If these gaps are not addressed, circular business models risk becoming nothing more than a buzzword in government policies and economic plans, without driving real change. This could mean missing out on the opportunity for the circular economy to boost global gross domestic product by $4.5 trillion by 2030. It could also jeopardize the $1 trillion in annual investment needed to meet the 2040 targets for reducing global plastic mismanagement. Between 2018 and 2023, global investments in plastics circularity totaled $190 billion, averaging $32 billion per year (The Circulate Initiative and International Finance Corporation, 2024).

Although government policies and business models are considered decisive for the circular transition, it is essential to explore and strengthen the public sector’s role in enabling this shift. Wasserbaur et al. (2022) identified three key dynamics between policymakers and business model designers: (i) entrepreneurs or managers can adjust their circular business models to benefit from existing policy frameworks; (ii) technological advancements can drive innovation in circular business models, prompting policymakers to adapt regulations; and (iii) policymakers can focus on the specific needs of circular business models to enhance their competitiveness. These interactions underscore the relevance of ongoing dialogue between policymakers and businesses to ensure that regulatory frameworks remain both supportive and flexible as the circular economy evolves. If Latin American governments act on this issue, they could help close the investment gap between emerging economies, which received $11 billion in 2023, and high-income economies, which secured $179 billion (The Circulate Initiative and International Finance Corporation, 2024).

The findings presented here are consistent with the limited but expanding body of literature on circular business models for plastics from a governmental perspective. Dijkstra et al. (2020) noted that most studies focus on recycling and bioplastics for value creation. Lit et al. (2024) stated that government support remains insufficiently targeted to help markets transition toward circularity. From the perspective of circular startups, van Opstal and Borms (2023) highlighted that diseconomies of scale and the predominantly linear structure of the sector are marked obstacles. von Kolpinski et al. (2023) elucidated internal challenges, including customer risk aversion, limited expertise in circular business modeling, and difficulty recognizing opportunities. Additionally, several countries have successfully incentivized circular models, such as the Netherlands and France, where EPR fees are eco-modulated based on recyclability or recycled content, or Sweden and Iceland, where mandatory producer responsibility and tailored fees support reuse and secondary materials adoption (Försterling et al., 2023; Laubinger et al., 2021).

### A Holistic Perspective to Address the Triple Planetary Crisis

This work points out that Latin America’s circular economy initiatives fail to adequately address the links between plastic management and the broader triple planetary crisis. Most countries have a general goal of reducing plastic pollution, but fail to specify types of negative impacts they aim to address. Moreover, the lack of specificity risks overlooking critical ecosystems (i.e., wetlands, mangroves, rivers, coral reefs, coastal zones, freshwater lakes, or marine habitats) that are disproportionately affected by plastic waste, thereby weakening the overall effectiveness of interventions. This paper calls on circular economy decision-makers to acknowledge these shortcomings and improve their approach to tackling the triple planetary crisis.

The circular economy’s role in global policy is increasingly tied to climate change. Paleari (2024) noted that the number of countries mentioning the circular economy in their nationally determined contributions to the Paris Agreement is increasing. Nonetheless, there is still no clear consensus on specific actions or monitoring systems to track how circularity contributes to climate change mitigation at the national level. A group of scientists recommends developing standardized indicators to highlight the potential benefits, trade-offs, and limitations of the circular economy in addressing climate change (Creutzig et al., 2024).

Biodiversity remains underemphasized compared to climate change in circular economy discussions. Buchmann-Duck and Beazley (2020) noted that biodiversity protection is rarely considered in circular economy theory and policy. They argue that while the circular economy cannot fully prevent environmental impacts due to humanity’s dependence on nature, it has the potential to reduce many of them. However, efforts to quantify how circular economy approaches affect biodiversity remain limited. Roberts et al. (2023) highlighted that companies across different sectors often lack an understanding of how the circular economy relates to biodiversity. Measuring the impact of circular practices on biodiversity remains complex, underscoring the need for more research and the development of reliable indicators and assessment methods. Nonetheless, some regions are beginning to bridge this gap. The European Union’s Biodiversity Strategy for 2030 explicitly connects circular design and reduced material extraction with habitat preservation. Similarly, Finland’s circular economy roadmap incorporates forest, soil, and aquatic ecosystem protection into its material-use targets (Hermoso et al., 2022).

The discussion of the triple planetary crisis and plastics in the circular economy gains greater relevance when considering their impact on human health. Several studies show that plastics affect human health through exposure to microplastics, plastic-associated chemicals, and environmental pollution (Winiarska et al., 2024). Beyond human health, recent research has provided evidence of the reduction in photosynthesis caused by microplastics in various ecosystems, which could threaten food security and link the fight against hunger to plastic pollution (Zhu et al., 2025).

Achieving plastic circularity is a complex challenge that impacts both human and environmental systems, requiring coordinated planning and resource management that moves beyond the siloed approach to the triple planetary crisis. Developing frameworks to apply the nexus approach and identify relevant indicators and target values to assess trade-offs and synergies has emerged as a promising solution. The nexus approach acts as a tool for integrating systems, engaging stakeholders, and exploring development pathways (Estoque, 2023). Another potential strategy is incorporating the planetary boundaries framework into circular economy initiatives (Erlandsson et al., 2023). Adopting these approaches could strengthen global policies. Estimates suggest that implementing stricter policies could reduce global gross domestic product by 0.5% by 2040 but would lead to significant improvements in environmental outcomes (OECD, 2024).

## Conclusions

This study provides a comprehensive overview of how Latin America manages plastics and the steps its governments are taking toward a circular economy for plastics. Although Latin American countries are not among the primary contributors to the global plastic crisis, current trends in the region raise concern. Some countries have shown a strong commitment to circularity, while others remain passive, with limited local action. However, several promising efforts are emerging, reflecting growing awareness of the need for circular plastic design, supported by research, standardization, and the promotion of certain circular business models.

Three immediate priorities are recommended for policymakers. They should strengthen regulations on microplastics and harmful additives, broaden the scope of circular models by considering their specific drivers and barriers, and adopt a more integrated and decisive approach to the triple planetary crisis. In this way, it is possible to build a coherent vision around three pillars: circular design as a foundation for reducing plastic waste from the beginning of the product’s life cycle, circular business models in plastics as a key enabler for driving low-carbon transitions, and combating the triple planetary crisis as an overarching environmental objective that should inform all circular economy initiatives. Governments increasingly have tools and evidence at their disposal to inform policy actions that guide the plastics industry toward decarbonization and circularity.

The novelty of this research lies in shedding light on the transition toward a circular economy for plastics through three interrelated dimensions: circular design, which has been moderately studied in other countries; circular business models, which remain underexplored in the Global South; and the triple planetary crisis, which requires more attention in circular economy debates worldwide. Furthermore, the findings of this work are relevant to regions facing similar development, governance, and environmental challenges. The emphasis on integrating circular design, evidence-based policymaking, and context-specific business models offers transferable lessons for other low- and middle-income regions, reinforcing the need for regionally adapted yet globally connected circular strategies.

The paper has some limitations. First, it relies on official documents, which may not capture the perspectives and contributions of key stakeholders like businesses, NGOs, or local communities. Second, by focusing only on national government commitments and excluding supra- or subnational circular economy plans, it overlooks initiatives at these levels that could strengthen national efforts. Third, some secondary data have unfortunately not been recently updated. Fourth, while the methodology provides a broad and previously unexplored regional perspective, it may not fully reflect the unique contexts and differences within individual countries.

Future studies may focus on critical research areas by: (i) developing circular economy-based selection frameworks for materials in plastics design; (ii) conducting a cost-benefit analysis to develop possible circular business cases in their various typologies, as well as identifying the cultural, political, commercial, and innovation drivers and barriers to these models in each country; and (iii) analyzing scenarios that integrate technological, economic, legal, and environmental aspects on how to achieve a circular economy for plastics that mitigates the worsening of the triple planetary crisis.

## Supplementary information

Supplementary material

## Data Availability

The data presented in this study are available on request from the corresponding author.
